# Development of the oral, mental, and sexual reproductive health assessment tool for adolescents in Nigeria

**DOI:** 10.3389/froh.2025.1592482

**Published:** 2025-10-30

**Authors:** Omorinola Adekemi Afolabi, Abel Nnamdi Chukwuemeka, Anita Mofiyinfolu Dabar, Richard Omoefe Oveh, Saheed Ademola Ibraheem, Nadia Adjoa Sam-Agudu, Maha El Tantawi, Abideen Olurotimi Salako, George Uchenna Eleje, Joanne Lusher, Oliver Ezechi, Moréniké Oluwátóyìn Foláyan

**Affiliations:** 1 Department of Social Development, Moshood Abiola Polytechnic, Abeokuta, Ogun, Nigeria; 2 Department of Public Health, Lead City University, Ibadan, Oyo, Nigeria; 3 Department of Information and Communication Technology, University of Delta, Agbor, Delta, Nigeria; 4 Scientific and Industrial Research Department, National Research Institute for Chemical Technology, Zaira, Nigeria; 5 International Research Center of Excellence, Institute of Human Virology, Abuja, Nigeria; 6 Department of Paediatrics and Child Health, University of Cape Coast School of Medical Sciences, Cape Coast, Ghana; 7 Department of Pediatrics, University of Minnesota Medical School, Minneapolis, MN, United States; 8 Faculty of Dentistry, Alexandria University, Alexandria, Egypt; 9 Oral Health Initiative, Nigerian Institute of Medical Research, Yaba, Lagos, Nigeria; 10 Africa Oral Health Network, Alexandria University, Alexandria, Egypt; 11 Faculty of Dental Medicine, Alamein International University, New Alamein City, Egypt; 12 Department of Obstetrics and Gynaecology, Nnamdi Azikiwe University, Awka, Nigeria; 13 Provost’s Group, Regent’s University London, London, United Kingdom; 14 Department of Child Dental Health, Obafemi Awolowo University, Ile-Ife, Nigeria

**Keywords:** adolescents, health screening, assessment tool, oral health, mental health, sexual and reproductive health

## Abstract

**Background:**

Adolescence is a critical developmental stage where oral, mental, and sexual and reproductive health are closely interconnected. However, these domains are often assessed in isolation, creating a gap in holistic adolescent health understanding and intervention. This study aimed to develop an integrated tool for assessing oral, mental, and sexual and reproductive health.

**Methods:**

A systematic literature review framework guided the study. Three dimensions—oral health, mental health, and sexual and reproductive health—were defined *a priori*. A structured search of PubMed and ScienceDirect identified relevant English-language articles and tools validated for use with adolescents in Nigeria. Deductive analysis was used for logical partitioning to identify items for domains and subscales. A preliminary questionnaire was drafted, organized into five sections: socio-demographics, oral health, mental health, sexual and reproductive health, and health service utilization. Items were matched with appropriate response scales.

**Results:**

Seventy-eight articles were identified, and 43 met the inclusion criteria. From these, domains and subscales were adopted to construct an 81-item tool. Section one contains 21 socio-demographic items. Section two covers mental health with five constructs: psychological distress (12 items), depression (nine), generalized anxiety (eight), suicide ideation (four), and risk factors (alcohol, tobacco, psychoactive substance use, and self-esteem). Section three measures sexual and reproductive health with 11 items on sexual debut and activity status. Section four assesses oral health with eight items on oral hygiene, self-reported oral problems, and oral habits. Section five includes two questions on health service utilization, covering general, dental, and psychiatric services.

**Conclusion:**

This integrated 81-item tool captures three interconnected aspects of adolescent health, offering potential to strengthen service integration for this population. Beyond practice, it provides a foundation for empirical research to advance multisectoral adolescent health approaches. Future work should focus on validating the tool across diverse adolescent populations.

## Introduction

Adolescents, defined as young people between 10 and 19 years of age (1), are at a critical stage of psychological, social, cognitive, and physical development (2). This is also a stage in life when habits are formed and health practices are developed, which can influence health outcomes at later stages of life (3–6). As such, adolescence is a distinctive stage of human development and a crucial time for establishing the foundations of good health. A strategic and integrated focus on the health and well-being of adolescents is therefore vital for optimal development in future health outcomes (7, 8).

Health-related habits that are formed during adolescence include oral health habits. Poor oral health can affect overall health, school performance, and social interactions (9). For adolescents in Nigeria, the high prevalence of oral diseases is a public health issue. Severe periodontal disease affects 25.1% of people 15 years and older (10), and dental caries affects about 22.9% (11). The large population of adolescents in Nigeria—more than one in four Nigerians are adolescents (12, 13) and 50% of the population is younger than 19 years (14)—suggests that the burden of oral diseases in this population is likely to be significant.

Also, of concern is the high burden of poor mental and sexual, and reproductive health among adolescents in the country. The prevalence of mental health disorders among adolescents ranges between about 10% and 37% (15–17), mainly in the form of depression, anxiety disorders, attention-deficit/hyperactivity disorder, and substance use disorder (18, 19). The high rates of unintended pregnancies, unsafe abortions, and sexually transmitted infections, including HIV, are also a concern, with reports of HIV prevalence being 14%–17%, and the prevalence of other sexually transmissible infections ranging from 29% to 48.8% (20–22).

The high prevalence of oral diseases, mental health challenges, and adverse sexual and reproductive health (SRH) outcomes among Nigerian adolescents is compounded by the significant interconnections between these domains. About 10%–37% of adolescents in Nigeria experience peculiar mental health challenges such as stress, depression, and anxiety (23–26). Poor access to sexual and reproductive health education increases the risks of early initiation of sex, teenage pregnancies, and sexually transmitted infections (27). About 11%—20.2% of adolescents initiate sex by 13 years (28, 29), 18.7%–22.9% of 15–19-year-olds are pregnant (30), and 13% to 95.1% experience sexual abuse (31). Poor oral health increases the risk for poor mental health (32) and vice versa (33). Poor oral health can also mediate poor sexual and reproductive health (34, 35).

These vulnerabilities converge because they share common social, economic, and behavioral determinants, such as family socioeconomic status, stigma, and health-seeking behaviors. This deep interrelatedness necessitates an integrated assessment tool to holistically capture these overlapping risks, which isolated evaluations inevitably miss. The current practice of using separate, siloed tools is clinically inadequate. Adolescents are often averse to referrals; when directed from one specialist to another (e.g., from oral to mental health services), many disengage due to stigma, cost, or mistrust, leading to fragmented care and missed intervention opportunities (36, 37). A unified OMSRH tool streamlines this process by enabling a “one-stop shop” assessment. This adolescent-friendly approach minimizes referral attrition, maximizes healthcare utilization, and is especially vital in resource-limited settings like Nigeria. By identifying co-occurring risks early, it facilitates the design of holistic interventions that address shared root causes, ultimately improving health outcomes and aligning with global strategies for integrated, adolescent-centered care (37).

There is currently no integrated instrument that measures these three areas of adolescents’ health despite their interconnectedness. The aim of this study was, therefore, to develop an integrated oral, mental, sexual, and reproductive health assessment tool for adolescents that will facilitate an inclusive and dynamic understanding of adolescent health in Nigeria.

## Methods

### Study design

**Figure 1:**
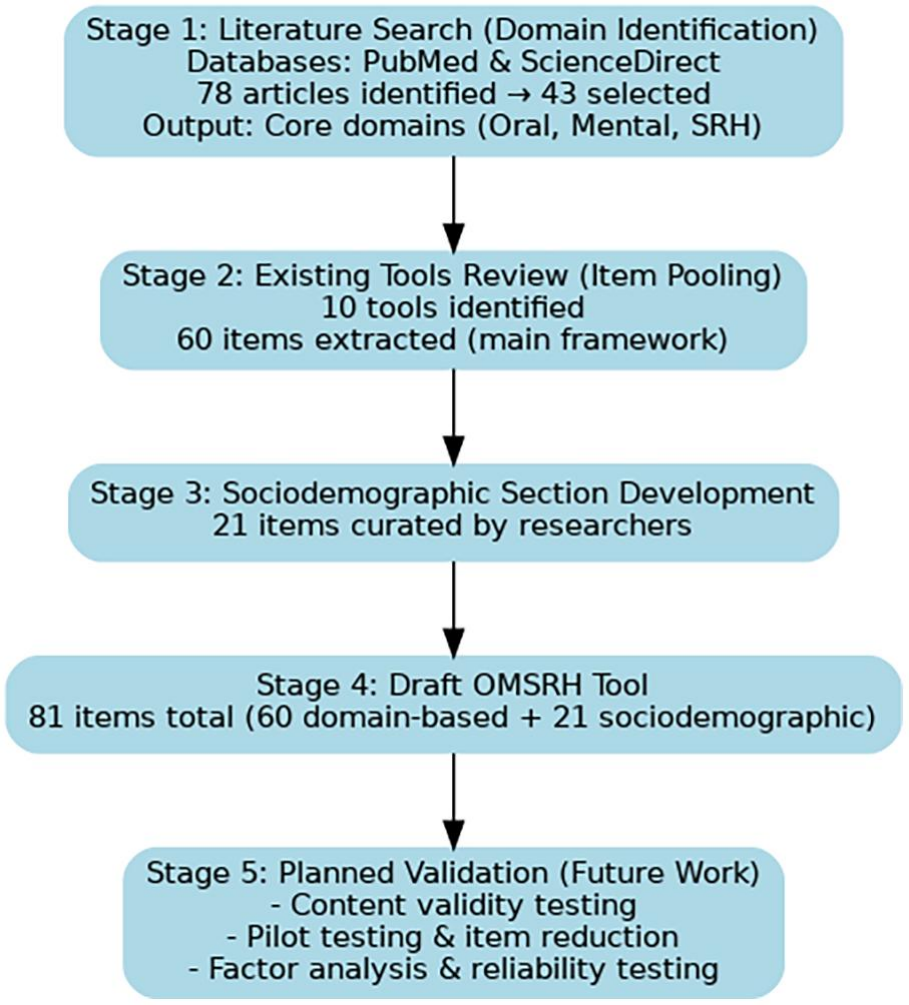
Flowchart summarizing the OMSRH tool development process.

A three-phased, nine-step mixed-methods approach proposed by Boateng et al. (36) was adopted for this study. This current study reports on the first of the three phases: conceptualization and item generation. The summary of the tool development process is presented in Figure 1.

### Domain identification

The study employed a systematic literature review framework, using a pre-defined and structured approach to identify and analyze relevant articles. The three key dimensions to be measured—oral health, mental health, and sexual and reproductive health—were defined *a priori*. A literature search was then conducted in PubMed and ScienceDirect to systematically identify articles or tools that conceptualized the measurement of these domains in English. There were no date restrictions. The identified articles were examined through thematic analysis to extract information on domains and subscales.

Articles were included if they addressed the conceptualization or measurement of at least one of the three dimensions and were designed for general health assessment in apparently healthy populations rather than for specific illnesses or unique contexts. Articles were excluded if they did not address any of the three dimensions or if they were highly specific to particular diseases or specialized populations.

A total of 78 articles were retrieved, with their full texts downloaded and reviewed for relevance. Of these, 12 articles were excluded for not addressing any of the three health dimensions, while 23 articles were excluded for being non-generic, as they were designed for specific illnesses or contexts. These were deemed unsuitable because the proposed tool aims to assess health among an apparently healthy adolescent population. Ultimately, 43 articles (38–80) were included in the study, and the domains and subscales statistically associated with any of the three key health dimensions were adopted for further development of the health assessment tool.

### Item generation

Next, the deductive method, also known as logical partitioning (81, 82), was used to identify items for the domains and subscales for the assessment tool. This approach to item generation in instrument design involves developing items based on existing theories, frameworks, or established concepts. Researchers first define the constructs they intend to measure and then generate items that align with these predefined concepts (81, 82). This method ensures theoretical consistency and validity, as items are directly linked to well-established literature and models. It is commonly used in structured assessments, ensuring that the instrument accurately reflects the intended constructs. However, it may limit the discovery of new dimensions not covered by prior theories (83).

### Item compilation

The measurement items were derived from validated tools and empirical studies, with most maintaining their original response scales, while a few were adjusted. The selected tools were those validated for use with adolescents in Nigeria, enhancing the likelihood that the measurement meets the five essential characteristics for ensuring the quality of construct measurement: consistent understanding, consistent administration or communication to respondents, clear communication of what constitutes an adequate answer, access to the information necessary for accurate responses, and respondents’ willingness to provide the correct answers required by the question at all times (83–87). Additional socio-demographic questions were formulated for the background section.

The preliminary questionnaire was then drafted, organizing pooled and formulated items into five sections: socio-demographics, oral health, mental health, sexual and reproductive health, and health service utilization.

## Results

These 43 included articles were only used to identify appropriate domains through which the dimensions of interest would be measured*.* Existing validated and unvalidated tools relevant to the domains identified from these articles were then reviewed*,* and items for the tool were generated from these. The existing tools identified at this stage include both globally recognized and validated ones, like GHQ-12, the Rosenberg self-esteem scale, PHQ-9, GAD-7, as well as unvalidated ones used in empirical studies among adolescents in Nigeria (23–25, 34, 43). Most items were retained verbatim, while some were rephrased. For clarity and cultural appropriateness to the Nigerian adolescent context. A summary of findings is presented in Table 1. The findings from the literature review are divided into oral health, mental health, and sexual and reproductive health.

**Table 1:** Summary of literature on domains for measuring oral, mental, and sexual reproductive health.

Dimension	Domains of measurement	Subscales	References
Oral health	Disability/handicap	Physical disability	(38, 39)
		Social disability	(38, 40)
		Psychological disability	(38, 41)
	Pain	Physical pain	(38)
	Limitation discomfort	Functional limitation	(38)
		Psychological discomfort	(42)
		Self-reported oral conditions	(23, 25, 42)
	Hygiene	Frequency of brushing, flossing	(44)
Mental health	General mental health	Anger, anxiety, emotion intensity, emotion regulation, energy, enjoyment of life, aspiration/goals, happiness/sadness, physical symptoms, relationship satisfaction, shame/guilt/disgust, self-worth	(45–57)
	Life satisfaction	Aspiration/goals, Autonomy, happiness/sadness, energy, enjoyment, self-worth, relationship satisfaction	(58–61)
	Quality of life	Resilience (ability to cope with pressure), Attention, Anger, concentration, Sleep quality, fear, worry, happiness/sadness, hyperactivity, loneliness, enjoyment, self-worth	(60–64)
	Symptoms	Aggression, anger, generalized anxiety, attention, concentration, depression, energy, enjoyment, happiness/sadness, hyperactivity, impulsivity, paranoid, physical symptoms, relationship satisfaction, self-harm, self-worth, suicide Ideation, withdrawal/loneliness	(51, 54–56, 65–68)
	Wellbeing	Resilience, Aspiration/goals, Concentration, Energy, enjoyment, happiness/sadness, loneliness, relationship satisfaction, self-worth	(49, 59, 60, 67, 68–76)
Sexual reproductive health	Sexual behaviour	Age at sexual debut	(77–80)
		Type of sexual partnership	(77–80)
		Use of protection during sex	(77–80)
	Health outcomes	Pregnancy	(77–80)
		Sexually transmitted infections	(77–80)

### Oral health

The literature review identified four key measurement domains (disability/handicap, pain, limitations/discomfort, and hygiene) and eight subscales. Physical, social, and psychological disabilities were recognized as essential subscales for understanding the broader impact of oral health conditions (38–41). Pain was primarily measured as physical pain (38), while functional limitations (38), psychological discomfort (42), and personal experiences of oral health problems (23, 25, 43) were measures of limitations/discomfort. In addition, providing insight into oral hygiene behaviours, such as frequency of brushing and flossing (44), was highlighted as a preventive indicator.

The oral health domain was structured around self-reported oral conditions, functional limitations, hygiene practices, diet, and self-reported health status, as identified in the review. The literature emphasized disability (physical, social, psychological), pain, and hygiene behaviours such as tooth brushing and flossing (38–41, 43, 44). In addition, dietary habits, particularly the frequency of refined sugar consumption, were recognized as significant factors influencing oral health (42, 43).

### Mental health

The review identified five measurement domains, emphasizing a multi-faceted approach to measuring mental health: general mental health, life satisfaction, quality of life, symptoms, and overall well-being. General mental health assessment includes emotional regulation, anxiety, anger, energy levels, self-worth, and relationship satisfaction (45–58). Life satisfaction was associated with autonomy, aspirations, and happiness (58–60), while quality of life incorporated resilience, attention, concentration, and sleep quality (59–61). Mental health symptoms, such as aggression, depression, anxiety, hyperactivity, impulsivity, and suicidal ideation, were identified as critical factors influencing adolescent health (51, 54–70). In addition, well-being was linked to resilience, aspirations, self-worth, and social relationships, aligning with broader mental health and quality of life frameworks. These frameworks include the Physical, Mental and Social Well-being Scale (PMSW-21) (50), the Life Orientation Test for optimism (60), the WHOQOL-SRPB BREF for spiritual and personal beliefs within quality of life (71), the Resilience Scale for Adolescents (READ) (72), the Well-Being Picture Scale (73), the Subjective Vitality Scale (74), the SPF-IL scale of social production functions (75), and the Questionnaire for Eudaimonic Well-Being (QEWB) (76).

For mental health, the literature highlighted general mental health indicators, including anger, anxiety, self-worth, and energy levels, which formed the basis for subscales such as ability to concentrate, worry, and confidence (45–58). Mental health symptoms, particularly depression, anxiety, and suicidal ideation, were cited as critical indicators of adolescent well-being (50, 54–58). Furthermore, mental health risk factors, such as tobacco use, alcohol consumption, psychoactive substance use, and self-esteem, were integrated based on their strong association with adolescent mental health outcomes (51, 60, 73–76).

### Sexual and reproductive health

The literature review categorized sexual and reproductive health into sexual behaviour and health outcomes. Sexual behaviour includes key indicators such as age at sexual debut, type of sexual partnerships, and use of protection during sex, which are essential for assessing risk behaviors and preventive practices (77–80). Health outcomes focus on pregnancy and sexually transmitted infections (STIs), reflecting critical consequences of sexual behaviors among adolescents (77–80).

The sexual and reproductive health domain was guided by studies focusing on sexual behaviour and health outcomes. The review emphasized age at sexual debut, type of sexual partnerships (including multiple and transactional partnerships), and condom use, which were included as essential subscales (78, 79). In addition, sexually transmitted infections (STIs) were identified as a critical health outcome, reflecting the risks associated with adolescent sexual behaviours (78, 80).

### Item measures

Based on the findings from the literature review, Table 2 outlines the final domains and subscales selected for the OMSHR Assessment Tool. The measurement items were derived from validated tools and empirical studies, with most maintaining their original response scales, while a few were adjusted. The selected tools were those validated for use with adolescents in Nigeria, enhancing the likelihood that the measurement meets the five essential characteristics for ensuring the quality of construct measurement: consistent understanding, consistent administration or communication to respondents, clear communication of what constitutes an adequate answer, access to the information necessary for accurate responses, and respondents’ willingness to provide the correct answers required by the question at all times (80–87). Additional socio-demographic questions were formulated for the background section. The preliminary questionnaire was then drafted, organizing pooled and formulated items into five sections: socio-demographics, oral health, mental health, sexual and reproductive health, and health service utilization.

**Table 2:** Selected domains and subscales for the oral, mental, sexual, and reproductive health assessment tool.

Dimension	Domain	Subscale
Background information	Demographics	Adolescent's demography
		Household socio-economic status
Oral health	Self-reported oral habits/conditions	Oral conditions, oral habits
	Functional limitation	
	Hygiene	Dental hygiene
	Diet	Frequency of consumption of refined sugars
	Self-reported health status	
Mental health	General mental health	Ability to concentrate, worry, and confidence
	Symptoms	Depression, anxiety, and suicide ideation
	Mental health risk factors	Tobacco use, alcohol consumption, psychoactive substance use, and self-esteem
Sexual reproductive health	Sexual behaviour	Age at sexual debut, type of sexual partnership (multiple sexual partnerships, transactional sex), use of protection during sex, sexually transmitted infections (STIs)
Health service utilisation	Type of healthcare service assessed, Barriers to healthcare service utilization	Frequency of healthcare service utilization, type(s) of healthcare service accessed, and barriers to healthcare service utilization

### Socio-demographics

This section aims to gather background information and family structure details. It includes personal data such as age, date of birth, gender identity, height, weight, school enrollment status, type of school, class (for in-school adolescents), education level, and occupation (for out-of-school adolescents). Most questions are open-ended, except for the secondary school type, which uses a four-point scale (Public Day, Public Boarding, Private Day, Private Boarding) to assess family socio-economic status. The second part focuses on family structure and socio-economic background.

### Oral health

Oral health was evaluated across five domains: self-reported oral health habits, functional limitation, oral hygiene, diet, and self-reported oral health status. Oral habits are indicators of physical oral health status and indicators of potential manifestations of co-occurring mental health issues; thus, they can capture overlapping vulnerabilities and provide a more holistic assessment of adolescent well-being. Self-reported oral health habits were assessed by asking whether participants engaged in any of eight behaviours that could impact oral health or overall well-being—digit or finger sucking, tongue sucking, tongue thrusting, lip sucking, lip biting, nail biting, object biting, and bruxism, with responses recorded on a dichotomous (yes/no) scale (25). Responses are on a dichotomous (yes/no) scale.

Functional limitation was evaluated with three items. The first item asked whether participants faced challenges performing any of the five mouth functions, with responses on a dichotomous (yes/no) scale. The second item asked respondents to rate the health status of their teeth, lips, tongue, oral mucosa, and jaws using a 5-point Likert scale, ranging from “excellent” to “poor.” The third item asked how much their overall life had been negatively affected by conditions of these oral parts, with responses on a 5-point Likert scale from “not at all” to “very much” (25).

Oral hygiene was assessed with one item that asked how often participants brushed their teeth. Item response is on a 5-response continuum ranging from “never or irregular” to “more than once a day” (23–25).

Diet was also assessed with an item that inquired about the frequency of consumption of sugar-containing snacks or drinks between main meals. Item response is also on a 5-response continuum ranging from “about 3or more times a day” to “occasionally, not every day” (23–25).

Self-reported oral health status involved asking about ten common oral health problems –tooth holes, tooth sensitivity, bleeding gums, bad breath, fractured or discoloured teeth, painful teeth, mouth ulcers, missing teeth, and no tooth problems, also rated on a dichotomous scale (yes/no), with the item and scale modified from (25).

### Mental health

Mental health was assessed through four domains: psychological distress, depression, generalized anxiety, and suicide ideation, chosen for their relevance to adolescents’ experiences.

Psychological distress was measured using the 12-Item General Health Questionnaire (GHQ-12), which includes items about recent experiences such as concentration, sleep, decision-making, and overall emotional well-being (55). The GHQ-12 is widely used to screen for mental health issues among different populations, both people with underlying medical conditions and in apparently healthy people (86–88), and is recognized for its unidimensional measure of psychological distress (89–91). The tool has been validated for use in Nigeria (92, 93), both in clinical and community settings. Studies in Nigeria (88, 94) have reported high internal consistency for the GHQ-12, with Cronbach's alpha values ranging from 0.78 to 0.92, indicating strong reliability. The optimal cutoff score for detecting psychological distress varies but commonly falls between 3 and 4 (92, 93). To assess its concurrent validity, the GHQ-12 was validated against structured clinical interviews such as the Structured Clinical Interview for Diagnostic and Statistical Manual of Mental Disorders (DSM) Disorders (SCID), showing good sensitivity and specificity in detecting psychological distress (92). Its sensitivity rates are above 85% (92) and specificity around 80%–90% (92, 93), indicating that the GHQ-12 is a reliable screening tool in Nigerian settings.

Depression was measured with items from the Patient Health Questionnaire (PHQ-9) (95), which assesses symptoms like lack of interest, sleep disturbances, low energy, and feelings of worthlessness. Responses are rated on a four-point scale, ranging from “not at all” to “nearly every day.” The PHQ has been validated for use in Nigeria in both clinical (among stroke patients (93) and community settings among university students (96). Studies have reported high Cronbach's alpha values (≥ 0.80) (96, 97), indicating strong internal consistency and reliability. This suggests that the PHQ-9 items are measuring the same construct (depression) consistently across respondents. Its test-retest reliability also demonstrated good stability over time in Nigerian samples, with high correlation coefficients (r = 0.80) in repeated measures (97). Factor analysis to assess its construct validity also confirmed a one-factor structure, aligning with the core concept of depression (96).

Generalized anxiety was measured with the GAD-7 scale, which includes items on nervousness, excessive worry, difficulty relaxing, and irritability, also rated on a four-point scale (98). Studies that assessed the psychometric properties of the GAD in Nigerian populations were not found.

Suicide ideation was assessed using the Suicide Behaviours Questionnaire—Revised (SBQ-R), which includes questions on lifetime and past-year suicidal thoughts, attempts, and the likelihood of future attempts (99, 100). The psychometric properties of the SBQ-R were tested among a population of students in tertiary institutions (101). The SBQ-R has shown strong construct validity among Nigerian respondents, through significant positive correlations with the HADS-Anxiety and Depression subscales, and the GHQ-12 (101). Cronbach's alpha for the SBQ-R items was 0.80. Receiver Operating Characteristics curve evaluation suggests that the best cut-off total score with the optimal sensitivity (0.882), specificity (0.875), and highest accuracy (0.879) was 8 in terms of identifying the students at high risk of suicide (101).

In addition, the tool evaluated mental health risk factors, including alcohol consumption, smoking, and psychoactive substance use, as well as self-esteem. Self-esteem was measured using the Rosenberg Self-Esteem Scale, which consists of ten statements about self-worth, with responses on a 4-point Likert scale to minimize response bias (56, 102).

### Sexual reproductive health

The tool assesses sexual reproductive health in two domains: sexual behaviour and health outcomes. Sexual behaviour is evaluated through lifetime experience of sexual intercourse, age at sexual debut, circumstances surrounding it, forms of intercourse (anal, vaginal, oral), and types of sexual engagement (transactional, multiple partnerships) in the past 12 months. It also measures the frequency of condom use during consensual sex and the protection used against sexually transmitted infections (STIs) in the last sexual encounter. The health outcome domain includes the incidence of STIs in the past six months and self-rated sexual health status, assessed on a six-point scale: Excellent, Good, Fair, Poor, Very Poor, and Don’t Know. The part of the tool was derived from the National HIV and AIDS and Reproductive Health Survey (NARHS) (103).

### Health service utilisation

The tool assessed health service utilization with two domains—types of healthcare service accessed and the barriers to utilization of healthcare service. The type of healthcare service assessed was measured with two items. The first item asked participants when they visited any of the healthcare professionals listed last time. Healthcare professionals listed in the item response are dentists, general practitioners, psychiatrists, and psychologists (25). Barriers to healthcare service utilization were measured with one item that asked participants for reasons why they have not visited any of the identified healthcare professionals in the past 6 months (25).

An 81-item tool was developed to assess adolescents’ oral, mental, and sexual reproductive health, comprising five sections. Section one includes 21 questions on socio-demographic variables. Section two evaluates mental health through four constructs: psychological distress (12 questions), depression (9 questions), generalized anxiety (8 questions), and suicide ideation (4 questions), along with six questions on mental health risk factors—alcohol, tobacco, and psychoactive substance use, and self-esteem. Section three addresses sexual and reproductive health with 11 questions covering sexual activity, age at sexual debut, types of sexual intercourse (oral, vaginal, or anal), sexual partnerships (multiple, transactional, heterogenous, gay/lesbian, others), condom use, sexual outcomes (STI incidence), and self-rated sexual health status. Section four assesses oral health through eight questions on oral hygiene, self-reported oral problems, oral habits, and functional limitations. Section five contains two questions on health service utilization, including general, dental, and psychiatric care. The instrument, which is currently at the conceptualization stage and has not been validated, can be found in Supplementary File 1.

Table 3 discusses the suggested scoring criteria for the instrument. The tool assesses adolescent well-being across mental health, sexual and reproductive health, and oral health, with sociodemographic and service utilization sections used only for descriptive stratification and not scored. Overall scores range from about 55 to 247, but interpretation relies on the profile of scores within domains rather than the composite number. The Mental Health Domain (44–180) is the most detailed, covering psychological distress (10–40), depressive symptoms (9–36), anxiety (7–28), suicidal ideation (4–16), risk behaviors (5–20), and self-esteem (10–40). Severity is graded, with high scores indicating greater problems, except for self-esteem, where higher is better. Notably, a score ≥3 on any suicidal ideation item requires urgent referral. The Sexual and Reproductive Health Domain uses risk indices rather than clinical cut-offs. Key measures include the Composite Sexual Risk Index, contraception/condom use (0–5, higher = riskier), and transactional sex/multiple partners (0–12, higher = greater vulnerability). Scores reflect relative levels of risk across behaviors. The Oral Health Domain (0–33) balances protective and risk factors: oral conditions (0–9, higher = more problems), hygiene habits (0–12, higher = better practices), diet/sugary snacks (0–4, higher = more risk), self-rated oral health (0–4, higher = poorer perception), and functional limitation (0–4, higher = more impairment). By synthesizing across domains, adolescents can be classified as low risk (normal scores throughout), moderate risk (mild to moderate severity in at least one domain), or high risk (severe findings in any domain, such as high suicidal ideation, severe depression/anxiety, extreme sexual health risk, or very poor oral health). High-risk profiles indicate the need for urgent and comprehensive intervention.

**Table 3:** Scoring framework for the OMSRH tool.

Domain/subscale	Items	Response options/scoring	Total score range	Interpretation/cut-offs
Sociodemographic information	Q101–Q121	Not scored (descriptive only)	–	Used for stratification by age, sex, SES, and location
Mental health				
Psychological distress (GHQ-like)	Q201–Q210	1–4 (higher = more distress; reverse-score Q210)	10–40	≤15 = Low; 16–23 = Moderate; ≥24 = High
Depressive symptoms (PHQ-like)	Q211–Q219	1–4 (higher = worse)	9–36	≤9 = Minimal;
				10–14 = Mild;
				15–19 = Moderate;
				≥20 = Severe
Anxiety symptoms (GAD-like)	Q220–Q227	1–4 (higher = worse)	7–28	≤9 = Minimal;
				10–14 = Mild;
				15–19 = Moderate;
				≥20 = Severe
Suicidal ideation	Q228–Q231	1–4 (higher = greater risk)	4–16	Any score ≥3 on any item = urgent referral flag
Risk behaviors	Q232–Q236	1–4 (higher = greater frequency/risk)	5–20	Report descriptively; higher = greater risk
Self-esteem (Rosenberg-like)	Q237–Q246	1–4 (reverse-score negatives; higher = better)	10–40	Higher = healthier self-esteem
Sexual and reproductive health				
Sexual activity & practices	Q301–Q310	Binary (Yes = 1, No = 0) or frequency-based (higher = riskier)	Variable	Composite sexual risk index: higher = more vulnerable
Contraception & condom use	Q311–Q315	Yes = 0 (protective), No = 1 (risk)	0–5	Higher = greater unprotected sex risk
Transactional sex & multiple partners	Q316–Q319	Frequency scored 0–3	0–12	Higher = greater vulnerability
Reproductive health experiences	Q320–Q324	Binary (Yes = 1, No = 0)	0–5	Report descriptively (clinical implications vary)
Oral health				
Oral conditions	Q401, Q404	Yes = 1 per problem	0–9	Higher scores = more conditions reported
Oral hygiene habits	Q402, Q405, Q406	Frequency (Never = 0, Daily = 4); reverse for poor habits	0–12	Higher scores = better hygiene
Diet (sugary snacks)	Q403	Frequency scale (higher = more risk)	0–4	Higher scores = worse diet quality
Self-rated oral health	Q407	0–4 (higher = poorer rating)	0–4	Higher scores = worse perceived oral health
Functional limitation	Q408	0–4 (higher = worse)	0–4	Higher scores = more limitations
Health service utilization	Q409–Q410	Report descriptively	–	Used to assess barriers to access
Composite OMSRH risk profile	All domains	Domain-specific scores summarized	–	Low risk = low across all domains;
				Moderate risk = moderate in at least 1 domain;
				High risk = severe/high in at least 1 domain

## Discussion

The study aimed to develop an integrated tool to assess oral, mental, and sexual reproductive health (OMSRH) among adolescents, addressing the interconnectedness of these health domains. The outcome was the creation of an 81-item tool divided into five sections, capturing a comprehensive picture of adolescent health by incorporating validated items from existing instruments and newly formulated questions. This tool represents a significant step toward integrating health assessments for adolescents, which could facilitate better service delivery and research in this population.

One of the strengths of the current study is that the tool measures multiple related dimensions of adolescent health, including socio-demographics, mental health, sexual and reproductive health, oral health, and health service utilization. This holistic approach ensures that the interconnectedness of these health domains is captured, which is crucial for understanding adolescent well-being. In addition. many items in the tool were adapted from well-established instruments, reducing the risk of construct underrepresentation (104) and construct-irrelevant variance (105) that can lead to the invalidation of the tool (106, 107). This enhances the tool's credibility and ensures that the constructs being measured are grounded in prior research. The tool also includes both dichotomous and Likert-scale response options, allowing for flexibility in measuring different constructs. The Likert-type response scales with five points have higher reliability, and are appropriate for use with the unipolar items measured in the instrument (108). This adaptability makes it suitable for diverse adolescent populations and various research settings. Furthermore, by combining oral, mental, and sexual reproductive health into a single tool, the instrument has the potential to streamline health assessments and promote integrated service delivery for adolescents, particularly in resource-limited settings like Nigeria.

Despite the study limitations, the development of this tool opens up multiple potential for improving adolescent health outcomes. The tool is still in the first phase of development. While the items have been generated and organized, the tool has not yet undergone content validation, cognitive interviews, or pilot testing. This means that its reliability and validity have not been established for any context or any population. Future steps for validation are discussed, enabling the tool to serve as a foundation for integrated health interventions that address the interconnected health needs of adolescents. The tool could also facilitate empirical research that explores the relationships between these health domains, providing valuable insights into the factors that influence adolescent well-being. Although the 81-item tool is also currently long, it is anticipated that the validation of this comprehensive item pool would lead to a shorter, more parsimonious, yet psychometrically sound instrument.

To realize this potential, the next steps should involve refining and validating the tool. This will include conducting a content validity and logical validity assessment, where an expert panel reviews the tool to confirm that the items are relevant, clear, and comprehensive. This process helps identify any gaps or redundancies in the tool, ensuring it accurately measures the intended constructs (106–111), while also providing preliminary evidence of the instrument's construct validity (112–116). In addition, conducting cognitive interviews with adolescents about the tool will help refine the tool's clarity, ensure that the questions are easily understood by the target population, improve the tool's usability, and reduce response bias (117, 118).

Furthermore, the developed tool needs to be pilot tested with a small sample of adolescents to evaluate its reliability using statistical measures such as Cronbach's alpha and Intraclass Correlation Coefficient (119, 120). This will provide initial insights into the tool's internal consistency and stability (110, 111, 114, 116). The tool's construct validity still needs to be determined through factor analysis and criterion analysis. Factor analysis will help confirm the tool's underlying structure, while criterion analysis will assess how well the tool correlates with other established measures of adolescent health (113–115).

Based on the findings from the pilot test and validity assessments, revisions should be made to refine the tool (113, 120). Following this, a large-scale validation study would be conducted to confirm the tool's reliability and accuracy across diverse adolescent populations. A minimum of 300–450 is recommended (121–123). Efforts would be made to reduce the number of items without compromising the tool's comprehensiveness—only parsimonious, functional, and internally consistent items would be ultimately included (116). This will enhance its practicality and ease of administration in real-world settings (124).

Finally, the tool needs to be evaluated through dimensionality testing (125–128) and confirmatory factor analysis (122, 126, 128) on a new sample. The finalized items from these tests can be used to generate scale scores for substantive analysis (82, 90). In addition, reliability (112, 114, 129) and tool validity (113–115, 130) assessments must be conducted. If the tool is to be applied in different cultural contexts, further adaptation may be required to ensure its relevance and appropriateness. This could involve modifying certain items or response options to better align with the cultural norms and health perceptions of the target population (131). This first version of the OMSRH tool has been intentionally designed as a health status, self-report instrument, suitable for use across diverse adolescent settings (schools, community centers, hospitals, and youth programs).

## Conclusion

The OMSRH is a newly designed tool that marks the first step in systematically measuring the oral, mental, sexual, and reproductive health needs of adolescents, thereby promoting an integrated approach for managing the critical health needs of adolescents. Its holistic approach, use of validated items, and potential for integrated service delivery make it a valuable resource for assisting in improving adolescent health outcomes. However, further development and validation are necessary to confirm its reliability and validity. Following further validation testing, this tool can serve as a powerful instrument for research, policy, and practice, ultimately contributing to better health and well-being for adolescents in Nigeria and beyond.

## Data Availability

The original contributions presented in the study are included in the article/Supplementary Material, further inquiries can be directed to the corresponding author.
