# Supplementary material for: Development of the oral, mental, and sexual reproductive health assessment tool for adolescents in Nigeria
Source: Front Oral Health. 2025 Oct 30;6:1592482. doi: 10.3389/froh.2025.1592482 (PMC12611828; doi:10.3389/froh.2025.1592482)
Supplement: Supplementary file 1 [file Table1.doc]

**ORAL, MENTAL AND SEXUAL REPRODUCTIVE HEALTH (OMSRH)**

# INDIVIDUAL INTERVIEW SCHEDULE

Questionnaire Identification Number |___|___|___|___|___|___|

**Introduction:** My name is Omorinola Adekemi Afolabi. I am working on a project to design a tool for screening adolescents (13 – 19 years) for oral, mental and sexual reproductive health of adolescents. The project is implemented by the Oral Health Initiative OHI), Obafemi Awolowo University, Ile Ife and Centre for Reproduction and Population Health Studies, Nigerian Institute for Medical Research, Lagos. The study is been led by Prof. Morenike Ukpong. This tool would implement a prototype oral health clinic where adolescent receive integrated oral, mental, sexual and reproductive health services. This tool is been developed as a screening tool. This tool will be essential in guiding future interventions and policies that promote holistic well-being among adolescents.

**Confidentiality and consent:** I am going to ask you questions some of which may be very personal. Your answers are completely confidential. Your name will not be written on this form. Your honest answers to these questions will help us better understand what the oral habits in this environment are and how we can work with parents of children who practice these oral habits to stop it. The information collected from you and people like you will help us make adequate plans for clinic and community of children who have oral habits. We would greatly appreciate your help in responding to this survey.

**Interviewer V**isit

|  | **Visit 1** | **Visit 2** | **Visit 3** |
| --- | --- | --- | --- |
| Date |  |  |  |
| Result |  |  |  |
| Interviewer |  |  |  |

**Name______________ Signature________**

**TIME INTERVIEW STARTED_______________**

Name of Coder____________________|___|___| Signature________________ Date______________

SECTION 1: ADOLESCENTS’ SOCIO-DEMOGRAPHIC

CHARACTERISTICS

| **No.** | **Questions and filters** | **Coding categories** | **Skip to** |
| --- | --- | --- | --- |
| Q101 | What was your age at your last birthday  **[COMPARE WITH Q102 IF NEEDED AND CORRECT Q101]** | Age in completed years [___|___] |  |
| Q102 | When were you born? (Date of birth): | Month [___|___]  Don’t know month ………..88  Year [___|___ [___|___]  Don’t know year ………..88 |  |
| Q103 | What is your sex at birth? | Male…………….1  Female………….2 |  |
| Q104 | What is your Gender identity | Male….………………………….1  Female………..…………………2  Transgender(Male)……………….3  Transgender (Female)……………..4  Non-Binary………………………..5  Genderqueer…………………….6  Genderfluid……………………….7  Prefer not to say…………………8  Others specify[­­­­_____________]…..99 |  |
| Q105 | What is your Height | [­­­­______________] |  |
| Q106 | What is your Weight? | [­­­­______________] |  |
| Q107 | Are you currently enrolled in a school? | Yes …………………1  No ………………….2 | **→Go to Q110** |
| Q108 | What type of school do you attend? | Public day school….……….1  Public Boarding School.……. 2  Private Day school……….. 3  Private Boarding School.…….. 4 |  |
| Q109 | What Class are you? | JSS1(Upper Basic 7)………………..1  JSS 2(Upper Basic 8)…………………2  JSS 3(Upper Basic 9)………………….3  SSS1…………………………….4  SSS2…………………………….5  SSS3………………………….6  Others specify[­­­­______________]…99 | **→Go to Q112** |
| Q110 | What level of education did you complete before leaving school? | JSS1(Upper Basic 7)………………..1  JSS 2(Upper Basic 8)…………………2  JSS 3(Upper Basic 9)………………….3  SSS1…………………………….4  SSS2…………………………….5  SSS3………………………….6  Others specify[­­­­______________]…99 |  |
| Q111 | What Vocation/occupation are you involved in? | [­­­­______________] |  |
| Q112 | How would you describe the area where you live? | Rural………….…1  Peri-urban ……...2  Urban …….….. 3 |  |
| Q113 | Religion | Islam…………. 1  Protestant.……. 2  Catholic……….. 3  Traditional.…….. 4  No religion ……. 5  Others specify[ ]…6  No Response………99 |  |
| Q114 | How important are religion and religious views to you? | Not important at all……..1  Fairly Important…………2  Important………………..3  Very important…………..4 |  |
| Q115 | Who do you currently live with? | Both Parents…………….1  Mother only…………….2  Father only……………..3  Mother and stepfather.....4  Father and step mother…5  Guardian…………..…….6  Cohabiting………….…..7  Roomate………………...8 |  |
| Q116 | What is your father’s/Guardian’s Occupation: | Skilled (self employed) ..……………....1  Unskilled………………..…………..2  Unemployed…………………...……3  Civil Servant …………………..…….4  Student………………………..…….5  Others specify[ ]…99 |  |
| Q117 | .What is your mother’s/Guardian’s Occupation | Skilled (self employed) ..……………....1  Unskilled………………..…………..2  Unemployed…………………...……3  Civil Servant …………………..…….4  Student………………………..…….5  Others specify[ ]…99 |  |
| Q118 | What is your father’s/Guardian’s highest level of   education. | No formal Education……….1  Quranic Education only……..2  Primary Education………….3  Secondary Education………4  Tertiary Education…………5 |  |
| Q119 | What is your Mother’s/Guardian’s highest level of education | No formal Education……….1  Quranic Education only……..2  Primary Education…………..3  Secondary Education………..4  Tertiary Education………….5 |  |
| Q120 | What is your birth order? | First born…………1  Second born……….2  Third born………….3  Fourth born………..4  Others specify[ ]..…99 |  |
| Q121 | How many are you in your family? | 2 people………..1  3 people………..2  4 people………..3  5 people………..4  6 people………..5  >6 people………6  Others specify[ ]..…99 |  |

**Section 2: Mental Health**

| **No.** | **Questions and filters** | **Coding categories** | **Skip to** |
| --- | --- | --- | --- |
| **Section B1: Psychological Distress** | | | |
| Q210 | Have you recently been able to concentrate on what you are doing? | Much less than usual…..1  Less than usual………..2  Same as usual……….3  Better than usual…….4 |  |
| Q202 | Have you recently lost much sleep over worry? | No not at all………1  Not more than usual…….2  Rather more than usual…..3  Much more than usual……4 |  |
| Q203 | Have you recently felt you were playing a useful part in things | Much less than usual………1  Less than usual……………..2  Same as usual……………..3  More so than usual……….4 |  |
| Q204 | Have you recently felt capable of making decisions about things? | Much less than usual……..1  Less than usual………….2  Same as usual……………3  More so than usual……….4 |  |
| Q205 | Have you recently felt constantly under strain? | No not at all…….1  Not more than usual………2  Rather more than usual…….3  Much more than usual……….4 |  |
| Q206 | Have you recently felt you couldn't overcome your difficulties? | No not at all……….1  Not more than usual……2  Rather more than usual…….3  Much more than usual………4 |  |
| Q207 | Have you recently been feeling unhappy and depressed? | No not at all…………….1  Not more than usual………2  Rather more than usual…….3  More so than usual ……….4 |  |
| Q208 | Have you recently been losing confidence in yourself? | No not at all………………..1  Not more than usual……….2  Rather more than usual……3  More so than usual……..4 |  |
| Q209 | Have you recently been thinking of yourself as a worthless person? | No not at all………………..1  Not more than usual……….2  Rather more than usual…….3  More so than usual……..4 |  |
| Q210 | Have you recently been feeling reasonably happy, all things considered? | Much less than usual…….1  Less than usual………….2  Same as usual……………3  More so than usual………4 |  |
|  | | | |
| **Section B2: Depressive Symptoms**  Over the last 2 weeks, how often have you experienced any of the following problems? | | | |
| Q211 | Little interest or pleasure in doing things | Not at all…………1  Several days………2  More than half the days….3  Nearly everyday………..4 |  |
| Q212 | Feeling down, depressed, or hopeless | Not at all………1  Several days…….2  More than half the days….3  Nearly everyday……..4 |  |
| Q213 | Trouble falling or staying asleep, or sleeping too much | Not at all…….1  Several days…….2  More than half the days…..3  Nearly everyday……….4 |  |
| Q214 | Feeling tired or having little energy | Not at all…….1  Several days…….2  More than half the days…..3  Nearly everyday……….4 |  |
| Q215 | Poor appetite or overeating | Not at all…….1  Several days…….2  More than half the days…..3  Nearly everyday……….4 |  |
| Q216 | Feeling bad about yourself or that you are a failure or have let yourself or your family down | Not at all…….1  Several days…….2  More than half the days…..3  Nearly everyday……….4 |  |
| Q217 | Trouble concentrating on things, such as reading the newspaper or watching television | Not at all…….1  Several days…….2  More than half the days…..3  Nearly everyday……….4 |  |
| Q218 | Moving or speaking so slowly that other people could have noticed. Or the opposite being so  fidgety or restless | Not at all…….1  Several days…….2  More than half the days…..3  Nearly everyday……….4 |  |
| Q219 | Thoughts that you would be better off dead, or of hurting yourself. | Not at all…….1  Several days…….2  More than half the days…..3  Nearly everyday……….4 |  |
|  | | | |
|  | | | |
| **Section B3: Generalized Anxiety Symptom**   Over the last two weeks, how often have you been bothered by the following problems? | | | |
| Q220 | Feeling nervous, anxious, or on edge | Not at all…….1  Several days…….2  More than half the days…..3  Nearly everyday……….4 |  |
| Q221 | Not being able to stop or control worrying | Not at all…….1  Several days…….2  More than half the days…..3  Nearly everyday……….4 |  |
| Q222 | Worrying too much about different things | Not at all…….1  Several days…….2  More than half the days…..3  Nearly everyday……….4 |  |
| Q223 | Trouble relaxing | Not at all…….1  Several days…….2  More than half the days…..3  Nearly everyday……….4 |  |
| Q224 | Being so restless that it is hard to sit still | Not at all…….1  Several days…….2  More than half the days…..3  Nearly everyday……….4 |  |
| Q225 | Becoming easily annoyed or irritable | Not at all…….1  Several days…….2  More than half the days…..3  Nearly everyday……….4 |  |
| Q226 | Feeling afraid, as if something awful might happen | Not at all…….1  Several days…….2  More than half the days…..3  Nearly everyday……….4 |  |
| Q227 | If you checked off any problems, how difficult have these problems made it for you to do your  work, take care of things at home, or get along with other people | Not at all…….1  Several days…….2  More than half the days…..3  Nearly everyday……….4 |  |
|  | | | |
|  | | | |
|  | | | |
|  | | | |
|  | | | |
| ***Section B4: Suicidal Ideation***   Please tick the number beside the statement or phrase that best applies to you | | | |
| Q228 | Have you ever thought about killing yourself? | No, Never………..1  Yes, it was just a brief passing  thought……………2  Yes, I have had a plan to kill myself once but did not try to do it…………..3  Yes, i have had a plan to kill myself and really wanted to die………………...…4 |  |
| Q229 | Have you ever attempted to kill yourself? | No, Never………..1  Yes, it was just a brief passing  thought……………2  Yes, I have had a plan to kill myself once but did not try to do it…………..3  Yes, i have had a plan to kill myself and really wanted to die………………...…4 |  |
| Q230 | How often have you thought about killing yourself in the past year? | Never……………1  Rarely (1 time).……2  Sometimes (2 times)…3  Often (3 - 4 times)……4  Very often (5 or more times)….5 |  |
| Q231 | How likely is it that you will attempt suicide one day? | Never………………1  Rather unlikely………..2  Unlikely…………..….3  Rather Likely………....4  Very Likely…………5 |  |
| ***Section B5: Mental Health Risk Factors***  **Consumption of Cigarettes, Alcohol and Psychoactive Substance(s)** | | | |
| Q232 | Do you smoke or use tobacco (cigarettes, cigars, chew, or e-cigarettes or vapes)? | No, Never……………1  No, i used to but i quit……...…..2  Yes, once a month or less………3  Yes, a few times (2 - 3) a month……..4  Yes, a few times (2 - 3) times a week…………………..5  Yes, once a day or more……….6 |  |
| Q233 | How often do you take alcohol? | Never…………1  Rarely (less than once a week)………………2  Occasionally (once a week)……3  Often (2 - 3 times) a week……..4  Always (everyday)…………….5 |  |
| Q234 | Some people have tried a range of different types of Psychoactive drugs **(Drugs that make a person feel high**). Which of the following, if any, have you tried? | Marijuana…………..1  Glue (Solvent)………2  Cocaine………………..3  Heroin……………….4  Prescription drugs…….5  Others (please specify)……..6 |  |
| Q235 | For which of the following reasons did you try any of the drug? | For Fun……….1  Out of Curiosity…….2.  Peer pressure………….3  To cope with stress…….4  To enhance sexual performance………….5  To enhance mental capacity…….6 |  |
| Q236 | How would you rate your current mental health? | Excellent…….1  Good………..2  Fair…………..3  Poor…………4  Very poor…….5  Don't know……6 |  |

| **No.** | **Questions and filters** | **Coding categories** | **Skip to** |
| --- | --- | --- | --- |
| ***Section B5: Mental Health Risk Factors - Self Esteem***  Please record the appropriate answer for each item, depending on whether you  1-Strongly agree,  2- agree, 3-disagree, or 4 -strongly disagree with it. | | | |
| Q237 | On the whole, I am satisfied with myself. | Strongly agree………….….1  Agree………….….2  Disagree………….….3  Strongly disagree………….….4  Don't know………….….5 |  |
| Q238 | At times I think I am no good at all. | Strongly agree………….….1  Agree………….….2  Disagree………….….3  Strongly disagree………….….4  Don't know………….….5 |  |
| Q239 | I feel that I have a number of good qualities. | Strongly agree………….….1  Agree………….….2  Disagree………….….3  Strongly disagree………….….4  Don't know………….….5 |  |
| Q240 | I am able to do things as well as most other people | Strongly agree………….….1  Agree………….….2  Disagree………….….3  Strongly disagree………….….4  Don't know………….….5 |  |
| Q241 | I feel 1do not have much to be proud of. | Strongly agree………….….1  Agree………….….2  Disagree………….….3  Strongly disagree………….….4  Don't know………….….5 |  |
| Q242 | I certainly feel useless at times | Strongly agree………….….1  Agree………….….2  Disagree………….….3  Strongly disagree………….….4  Don't know………….….5 |  |
| Q243 | I feel that I'm a person of worth. | Strongly agree………….….1  Agree………….….2  Disagree………….….3  Strongly disagree………….….4  Don't know………….….5 |  |
| Q244 | I wish I could have more respect for myself | Strongly agree………….….1  Agree………….….2  Disagree………….….3  Strongly disagree………….….4  Don't know………….….5 |  |
| Q245 | All in all, I am inclined to think that I am a failure. | Strongly agree………….….1  Agree………….….2  Disagree………….….3  Strongly disagree………….….4  Don't know………….….5 |  |
| Q246 | I take a positive attitude toward myself | Strongly agree………….….1  Agree………….….2  Disagree………….….3  Strongly disagree………….….4  Don't know………….….5 |  |

**Section C: Sexual Reproductive Health**

| **No.** | **Questions and filters** | **Coding categories** | **Skip to** |
| --- | --- | --- | --- |
| Q301 | Have you ever had any form of sexual intercourse | Yes……….1  No………..2 |  |
| Q302 | At what age did you first have sexual intercourse if ever? | _________ |  |
| Q302 | What was the circumstances around your first sexual intercourse? | In love…………1  Having fun………2  Peer pressure……..3  To obtain money or other favours……………4  Forced……………5 |  |
| Q304 | Which of these forms of sex have you ever been involved in the last 12 months? | Vaginal……………1  Oral………2  Anal…………3 |  |
| Q305 | In the past 12 months, have you ever had sex in exchanged for money, a place to stay, or material goods etc.? | No, never……………1  Yes, Occasionally……2  Yes, frequently……..3  Yes, always………4 |  |
| Q306 | How many sexual partners have you had in the past 12 months? | Number of male ---------------------  Number of female--------------------  Number of people who use another term (please specify term) |  |
| Q307 | The last time you had consensual sexual intercourse; did you or your partner use a condom? | Yes……………….1  No……………..2 |  |
| Q308 | The last time you had sexual intercourse, what kinds protection against sexually transmitted  disease(s) or (pregnancy) did you use? (Tick all that apply)? | No precaution………………1  External (male) condom……..2  External female) condom……3  Dental dam…………….4 |  |
| Q309 | In the past 6 months, have you experienced any form of vaginal discharge ? | Yes……………………..1 No……………..2 |  |
| Q310 | In the past 6 months, have you had an infection that is spread by having sex? (Like herpes, gonorrhea, chlamydia, genital warts, pelvic inflammatory disease, HIV, syphilis? | Yes……………1  No…………….2 |  |
| Q311 | How would you rate your current sexual health? | Excellent………..1  Good…………..2  Fair……………..3  Poor……………4  Very poor………5  Don't know…….6 |  |

**SECTION D: ORAL HEALTH**

| **No.** | **Questions and filters** | **Coding categories** | | **Skip to** | |  |
| --- | --- | --- | --- | --- | --- | --- |
| Q401 | Do you have any of the following oral health problems? | Yes………1  No……….2  If Yes, please specify;  Hole in tooth…….1  Sensitive tooth……2  Bleeding gums…….3  Bad Breath………..4  Fractured tooth……5  Discoloured tooth…6  Painful tooth……….7  Mouth ulcers……….8  Missing tooth………9  No tooth problem…..10 | |  | |  |
| Q402 | How often do you brush your teeth? | Never or Irregular………1  Once a week……………2  A few times (2 - 3) a week…3  Once a day……………….4  More than once a day……..5 | |  | |  |
| Q403 | How often do you consume sugar-containing snacks or drinks between main meals | About 3 times a day or more,…..1  About twice a day,……….2  About once a day,………..3  occasionally, not every day,….4  Rarely or never eat between meals……………………..6 | |  | |  |
| Q404 | Do you have challenge(s) performing any of the following functions with your mouth? | Eating………1  Speaking………2  Cleaning your mouth…..3  Smiling……………….4  Closing your mouth………5 | |  | |  |
| Q405 | How often do you floss your teeth? | Irregular or Never…………1  Once a week………………2  A few (2 -3) times a week……3  Once a day…………………4  More than once a day………..5 | |  | |  |
| Q406 | Do you have any of these oral habits | Digit and finger sucking……….1  Tongue sucking……………….2  Tongue thrusting…………….3  Lip sucking………………….4  Lip biting…………………..5  Nail biting…………………..6  Object biting………………..7  Bruxism………………………8 | |  | |  |
| Q407 | How you would describe health status of the following oral parts of your body (0) ‘excellent’; (1) ‘very good’; (2) ‘good’; (3) ‘fair’, and (4) ‘poor | | **Oral Parts** | **1** | **2** | **3** | | **4** | | **1** | | **2** | | **3** | | **4** | | | --- | --- | --- | --- | --- | --- | --- | --- | --- | --- | --- | --- | --- | --- | --- | | Teeth |  |  |  | |  | |  | |  | |  | |  | | | Lips |  |  |  | |  | |  | |  | |  | |  | | | Tongue |  |  |  | |  | |  | |  | |  | |  | | | Oral Mucosa |  |  |  | |  | |  | |  | |  | |  | | | Jaws |  |  | |  | |  | |  | |  | |  | |  | | |  | |  |
| Q408 | How much has your overall life been negatively affected by the conditions of the following  oral parts:  ‘not at all’; (1) ‘very little’; (2) ‘somewhat’ (3) ‘a lot’; and (4) ‘very much’ | | **Oral Parts** | **1** | **2** | **3** | | | **6** | **4** | | **1** | | | --- | --- | --- | --- | --- | --- | --- | --- | --- | --- | --- | | Teeth |  |  |  | | |  |  | |  | | | Lips |  |  |  | | |  |  | |  | | | Tongue |  |  |  | | |  |  | |  | | | Oral Mucosa |  |  |  | | |  |  | |  | | | Jaws |  |  | |  |  | | |  | |  | | |  | |  |
| **Section E: Health Service Utilization** | | | | | |  |
| Q409 | .   When was the last time you visited any of the following healthcare professional  1. Less than 6 months ago  2.   6 months to 1 year ago  3.   1–2 years ago  4.   2–5 years ago  5.  Over 5 years ago  6.     I cannot remember   7.    Never | | **Healthcare Professional** | **1** | **2** | **3** | **4** | **5** | **6** | |  | |  | | | **1** | | | **2** | | | **3** | | | **4** | | | **5** | | | **6** | | | **7** | | | | --- | --- | --- | --- | --- | --- | --- | --- | --- | --- | --- | --- | --- | --- | --- | --- | --- | --- | --- | --- | --- | --- | --- | --- | --- | --- | --- | --- | --- | --- | --- | --- | --- | --- | | Dentist |  |  |  |  |  |  | |  | |  | | |  | | |  | | |  | | |  | | |  | | |  | | |  | | | | General practitioner (Doctor) |  |  |  |  |  |  | |  | |  | | |  | | |  | | |  | | |  | | |  | | |  | | |  | | | | Psychiatrist |  |  |  |  |  | |  | |  | | |  | | |  | | |  | | |  | | |  | | |  | | |  | | |  | | Psychologist |  |  |  |  |  | |  | | | |  | | |  | | |  | | |  | | |  | | |  | | |  | | |  | | | 7 | |  | |
| Q410 | What has inhibited you from visiting any of the following professional healthcare practitioners  in the past | No health problem…………1  No availability of funds……..2  Do not know where to find on…….3  Feeling of lack of control with dentist…………….4  Treatment procedure too invasive…….5  Dental procedure will make me gag………..6  Reminds me of the experience of assault……………7  No reason…………8 | |  | |  |

**FINAL REMARKS ON THE QUESTONNAIRE**

**INTERVIEW CLOSING TIME………………………….**

**DATE………………………………………………………..**

##### LANGUAGE (s) THAT THE INTERVIEW WAS CONDUCTED …………………………….…………

**INTERVIEWER’S COMMENTS………………………………………………..**

**………………………………………………………………………………………**

**………………………………………………………………………………………**

**………………………………………………………………………………………**

**Name……………………………………………**

**Signature/Date…………………………………………..**
